# The Association Between Parental Phubbing and Preschoolers’ Excessive Electronic Media Use: The Chain Mediating Role of Parent–Child Attachment and Self-Control

**DOI:** 10.3390/bs16010121

**Published:** 2026-01-15

**Authors:** Qiong Zhao, Yanrong Fan, Kuai Song, Zhengyi Wang, Zongkui Zhou

**Affiliations:** 1 Key Laboratory of Adolescent Cyberpsychology and Behavior (CCNU), Ministry of Education, School of Psychology, Central China Normal University, Wuhan 430079, China; zhao.q@mails.ccnu.edu.cn (Q.Z.);; 2 Center for Mental Health Education of College Students, Zhengzhou University of Industrial Technology, Zhengzhou 451100, China; 3 School of Preschool Education, Hubei Preschool Teachers College, Ezhou 436032, China; 4 Mental Health Education Center, Wuhan Institute of Technology, Wuhan 430205, China

**Keywords:** parental phubbing, preschooler, excessive electronic media use, parent–child attachment, self-control

## Abstract

In the current digital age, children are exposed to electronic media at an increasingly early age. The issue of excessive electronic media use has become a significant risk factor affecting the healthy development of young children. To examine the association between parental phubbing and preschoolers’ excessive electronic media use, as well as the underlying mechanism—mediating roles of parent–child attachment and self-control, 758 parents of preschoolers were recruited to complete a set of scales. The results show that: (1) Parental phubbing was positively associated with preschoolers’ excessive electronic media use; (2) Parent–child attachment and self-control significantly mediated this relation, which contains three mediating pathways—the mediating effects of parent–child attachment and self-control, as well as their chain mediating effect. The study revealed the psychological mechanisms between parental phubbing and preschoolers’ excessive electronic media use, providing suggestions for the prevention and intervention of excessive electronic media use among preschoolers.

## 1. Introduction

In the current digital society, with the popularization and development of digital media devices, the age of contact and use of electronic devices is constantly advancing. The proportion of preschoolers accessing the internet also continues to rise. Moreover, the later they are born, the higher the proportion of preschoolers accessing the Internet (The Central Committee of the Communist Youth League of China’s Department for the Protection of Youth Rights, 2023). Young children rapidly acquire personal devices: 40% have tablets by age 2, rising to 58% by age 4. Nearly a quarter own cellphones by age 8. Children under 8 average 2.5 h of daily screen time, increasingly on short-form video platforms. One in five children use devices for comfort, meals, or sleep, which fuels significant parental concerns about screen time (Common Sense Media, 2025). However, the World Health Organization recommends that children aged 1–4 years should not spend more than one hour per day on screen time (World Health Organization, 2019), with no single session exceeding 15 min (Ministry of Education of the People’s Republic of China, 2018). There is a significant gap between the current use of electronic media by young children and official recommendations. The problem of excessive electronic media use has become increasingly prominent and has become an important risk factor affecting the healthy growth of young children in public health and developmental research (World Health Organization, 2019; Hinkley et al., 2014). Physiologically, the excessive use of electronic media among young children has been associated with reduced and disrupted sleep (Rapoport et al., 2019), obesity (Sisson et al., 2010), and developmental delays (Tamana et al., 2019). Psychologically, it is associated with attention deficits (X. Li et al., 2021), hyperactivity (Poulain et al., 2018), impaired social skill development (Jericho & Elliott, 2020), depression and anxiety (Fors & Barch, 2019), and aggression (Carson & Janssen, 2012).

Against this background, the influencing factors of preschoolers’ electronic media use have been the focus of relevant research. Research indicates that children’s electronic media use correlates with sleep duration (Lan et al., 2020), household chaos (Emond et al., 2018), physical environment, household income, parental education, screen time monitoring, and parental self-efficacy (Bjelland et al., 2015; Jago et al., 2013; Nagata et al., 2022; Pyne et al., 2025; Veldman et al., 2023). Taken together, these findings demonstrate that familial factors play a significant role in shaping preschoolers’ electronic media use. According to family systems theory and social learning theory, the family is a dynamic and interdependent system in which behavioral patterns, rules, roles, and emotions influence all members (Bowen, 1993). Family media norms have a hidden influence on children (Lauricella & Cingel, 2020). Parents’ screen usage habits and attitudes may be important factors influencing young children’s use of electronic media. Most of the existing research has concentrated on the impacts of excessive electronic media use on adolescents, while paying relatively little attention to preschoolers. Preschoolers exhibit developmental characteristics including strong attachment needs, immature self-regulation and executive functions (Best & Miller, 2010), a high tendency to imitate parental behavior, and a significant reliance on adult co-regulation for emotional management (Paley & Hajal, 2022). High screen exposure patterns during the preschool years may persist into the middle childhood period and even adolescence, carrying long-term implications (Yang et al., 2024). The physiological immaturity of preschoolers makes them significantly more sensitive to non-verbal cues than adolescents (Ding et al., 2018). Based on the above empirical findings and theoretical perspectives, this study aims to examine the association between parental phubbing and preschoolers’ excessive electronic media use and its internal mechanisms. The study will provide empirical support and intervention guidance to promote the reasonable use of electronic media by preschoolers.

### 1.1. Parental Phubbing and Preschoolers’ Excessive Electronic Media Use

Phubbing refers to the behavior of individuals who focus on using their mobile phones during interpersonal communication, thereby neglecting and ignoring others (Roberts & David, 2016). Phubbing represents social exclusion, reduces the quality of communication, and even interrupts communication, affecting the development and deepening of interpersonal relationships (Roberts & David, 2016; X. Wang et al., 2020). Parental Phubbing refers to the phenomenon of parents frequently using mobile phones, tablets, and other electronic devices during parent–child interactions, thereby neglecting communication and interaction with their children (Ding et al., 2018; X. Wang et al., 2020). A survey found that 52% of parents use their phones while spending time with their children or caring for them (Jiang et al., 2021). Observational studies further show that during meals, approximately 80% of parents repeatedly look at their phones, interrupting interaction with their children. When looking after children at playgrounds, 59% of parents use their phones and ignore their children’s need for attention (Radesky et al., 2014b). Based on family systems theory and social learning theory, the family functions as an interconnected system rather than a collection of independent individuals, such that parents’ media-related behaviors shape children’s behavioral patterns within the family context (Bandura, 1977; Y. Zhang et al., 2025). Children learn by observing and imitating the behavior of significant others, especially their parents, and they regard the observed behavior patterns as their behavioral norms (Bandura, 1977; Bae & Nam, 2023). Consistent with this perspective, empirical research has demonstrated that parents’ mobile phone use is positively associated with children’s digital media use (Veldman et al., 2023). The intergenerational transmission posits that parents’ behaviors, values, and cognitive patterns are passed on to their children through prolonged interaction (Bae & Nam, 2023; Gong et al., 2022). This transmission is not a simple replication, but rather gradually formed through observation, imitation, interaction, and reinforcement (Madden et al., 2015; Van Ijzendoorn, 1992). The transfer is implicit, continuous, and frequently occurs unconsciously (Mun, 2024). Parental phubbing significantly predicts problematic mobile phone use and internet addiction among minors (Niu et al., 2020). The higher the parents’ mobile phone dependence, the more likely their children are to develop mobile phone addiction (Ding et al., 2018; Hong et al., 2019; Y. Li et al., 2025). Taken together, prior theoretical frameworks and empirical findings consistently suggest that parental phubbing constitutes a salient family risk factor for children’s excessive digital media use. Based on this, this study puts forward the hypothesis H1: Parental phubbing was positively associated with preschoolers’ excessive electronic media use.

### 1.2. Parent–Child Attachment as a Mediator

Parent–child attachment usually refers to an emotional bond established between caregivers and infants or young children through repeated interactions (Armsden & Greenberg, 1987). Parent–child attachment is mainly influenced by factors such as parent–child interaction patterns (W. Wu et al., 2022), parenting styles (Hou et al., 2022), family environment (Deng et al., 2013), and family functioning (Y. Wang et al., 2021). Parents’ sensitivity to their young children’s needs is central to the formation of attachment relationships (Armsden & Greenberg, 1987; Kildare & Middlemiss, 2017). Parents’ positive responses to their young children’s various signals, behaviors, and needs help establish trust and secure parent–child attachment. According to the substitution hypothesis, empirical studies suggest that technological distractions such as parental phubbing increase the likelihood of distraction during parent–child interactions (Meeus et al., 2021). This phenomenon partially replaces the communication and interaction between parents and children. As a result, there is reduced parent–child interaction, especially nonverbal interaction, such as eye contact, smiling, and gestures (Meeus et al., 2021). A decrease in parent–child interaction may make it more difficult for young children to perceive their parents’ emotional support and attention, thereby reducing the quality of parent-child interaction (Jiang et al., 2021; Kildare & Middlemiss, 2017). When parents engage in media tasks while looking down, they respond to their children’s attention-seeking behaviors in a harsher, stricter, and indifferent manner (Radesky et al., 2014b). Preschoolers who frequently perceive parental phubbing experience greater emotional distance and reduced feelings of belonging, which has been associated with a higher likelihood of insecure parent–child attachment (Jiang et al., 2021; Xu & Xie, 2023).

Attachment theory emphasizes that the quality of parent–child relationships has a significant predictive effect on children’s development. A good parent–child attachment relationship can promote the formation of a sense of security and trust, while a poor parent–child attachment is an important risk factor for problem behaviors in preschoolers (Boniel-Nissim & Sasson, 2018). Studies have shown that parent–child attachment is closely related to problematic internet use among children (Xie et al., 2019). Negative parent–child interactions, including low responsiveness and emotional indifference, are positively correlated with an elevated frequency of children’s media use (Sampasa-Kanyinga et al., 2020). Parental phubbing causes young children to experience emotional rejection, resulting in unmet psychological needs such as belonging and autonomy (Xu & Xie, 2023). This may trigger a “compensatory effect”, prompting young children to seek alternative satisfaction through electronic media use (S. Chen et al., 2023; Niu et al., 2020; Xie et al., 2019). Taken together, existing theoretical frameworks and empirical findings consistently indicate that parental phubbing undermines parent–child attachment, which in turn increases the risk of excessive electronic media use in young children. Based on this, this study puts forward the hypothesis H2: Parent–child attachment mediates the relationship between parental phubbing and preschoolers’ excessive electronic media use.

### 1.3. Self-Control as a Mediator

Self-control is the ability to regulate one’s behavior and emotions effectively when faced with challenges or temptations. Its development depends heavily on parenting styles (Inzlicht et al., 2014). As the earliest social environment for young children, the behavior of parents influences the formation and development of self-control (Bunch et al., 2018). According to the self-control resource model Inzlicht et al., 2014), self-control depends on limited psychological resources. Empirical evidence suggests that long-term stress (such as emotional neglect or lack of guidance) will continuously deplete these regulatory resources, leading to a subsequent loss of behavioral control (Stockdale et al., 2018). On the one hand, parental phubbing may weaken emotional bonds, causing young children to remain in a state of chronic stress due to emotional neglect (David & Roberts, 2017; Kildare & Middlemiss, 2017). This situation accelerates the depletion of young children’s self-control resources and reduces their motivation for self-regulation and self-management (Stockdale et al., 2018), thereby increasing impulsive behavior (Bunch et al., 2018). On the other hand, parental phubbing can lead to a lack of effective behavioral supervision and guidance for young children. Research has indicated that effective external guidance helps children develop strategic resource reserves (Pinquart, 2017), whereas insufficient parental supervision impedes children’s internalization of necessary self-control strategies (X. Zhang et al., 2020), thereby affecting the formation of their self-control abilities.

Self-control theory argues that self-control plays a key role in preventing and responding to undesirable behaviors (Gottfredson & Hirschi, 1990). Multiple studies have confirmed that self-control plays an important role in children’s digital behavior regulation (Qiu et al., 2022; Lu et al., 2025). For instance, individuals with high self-control exhibit significantly reduced dependence on smart devices (Qiu et al., 2022), and self-control training programs effectively reduce children’s screen time (Lu et al., 2025). These findings support the notion that self-control failure in preschool children directly predicts problematic smartphone use (Mancinelli et al., 2022). It is worth noting that the role of self-control is independent, even after controlling for family variables, low self-control significantly increases the risk of addiction (Chua, 2019; S. Chen et al., 2025). Based on this, this study proposes hypothesis H3: Self-control mediates the relationship between parental phubbing and preschoolers’ excessive electronic media use.

### 1.4. The Chain Mediating Effect

According to self-control theory, a good parent–child attachment is the key prerequisite for the development of children’s self-control ability (Gottfredson & Hirschi, 1990). Secure attachment influences children’s behavioral control capabilities by shaping their cognitive modes of self and environment. On the one hand, the quality of parent–child attachment positively predicts the self-control levels of young children (Meldrum et al., 2012). Especially in multigenerational families, the mother-child attachment significantly promotes the development of young children’s self-control abilities (X. Li et al., 2024). On the other hand, secure attachment can enhance the internalization of parenting norms (Alvarez-Rivera et al., 2017), enabling children to more actively transform their parents’ values into their behavioral guidelines (M. Wu et al., 2023). This process is particularly critical in the digital context, as impaired interaction—such as that caused by parental phubbing—may hinder the internalization of these norms (Y. Sun et al., 2022). It is worth noting that interventions aimed at improving the quality of parent–child relationships have been demonstrated to be effective in enhancing the self-control capabilities of minors (Brody et al., 2005). Integrating these perspectives, parental phubbing may first disrupt the attachment, which in turn compromises the regulatory resources needed for self-control, eventually leading to excessive media use. Based on this, this study proposes hypothesis H4: Parent–child attachment and self-control play a chain mediating role between parental phubbing and preschoolers’ excessive electronic media use.

**Figure 1:**
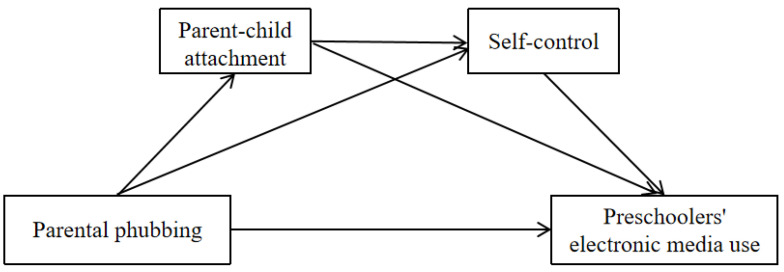
Research model.

Based on family systems theory, social learning theory, and attachment theory, the present study aims to explore the association between parental phubbing and preschoolers’ excessive electronic media use. Furthermore, a chained mediation model (Figure 1) was constructed to examine the mediating roles of parent–child attachment and self-control.

## 2. Methods

### 2.1. Participants and Procedure

A convenient sampling method was adopted to recruit parents of children aged 3–6 years in 8 kindergartens (including public, private, and government-subsidized kindergartens) in central China. The final sample size of 758 was determined by the study design and the availability of participants within the target population. The average age of the participating parents was 34.77 ± 4.68 years, including 150 fathers (19.79%) and 608 mothers (80.21%). Among them, 150 parents (19.79%) had a high school education or below, 180 (23.75%) had an associate degree, 330 (43.54%) had a bachelor’s degree, and 98 (12.93%) had a master’s degree or higher. The average age of the preschoolers was 4.12 ± 1.18 years, including 380 boys (50.13%) and 378 girls (49.87%), with 398 only children (52.51%) and 360 non-only children (47.49%). Socioeconomic status (SES) was assessed using a composite index of parental education level and monthly household income, consistent with common operationalizations of SES in developmental research (Bradley & Corwyn, 2002). In the participants of this study, all participants owned smart devices and permitted their children to have some exposure to electronic media.

The study was conducted in compliance with the Declaration of Helsinki and approved by the Ethical Institutional Review Board at the authors’ university. At the same time, this study was conducted with the informed consent of school leaders and parents. Before the formal investigation, participants were informed about the confidentiality of this study and their right to opt out at any time.

### 2.2. Measures

#### 2.2.1. Parental Phubbing

This study adopted the Parent Phubbing Scale (Zu et al., 2022), a unidimensional measure consisting of 9 items, such as “When I am out playing with my child, I play with my phone.” Responses were rated on a 5-point Likert scale (1 = never, 5 = always). A global mean score was calculated by averaging all items, with higher scores indicating a higher frequency of parental phubbing. The Cronbach’s α coefficient of this scale in the present study was 0.75.

#### 2.2.2. Preschoolers’ Excessive Electronic Media Use

This study used the Preschoolers’ Electronic Media Use Scale (P. G. Li, 2018), which consists of 20 items, such as “The child has reduced outdoor play due to electronic media use.” The scale comprises four dimensions: time management, interpersonal and health problems, life conflict, and emotional experience. Responses were rated on a 5-point Likert scale (1 = never, 5 = always). In the present study, item scores were summed and then averaged to create a global mean score, with higher scores indicating more excessive electronic media use among preschoolers. The Cronbach’s α coefficient of this scale in the current sample was 0.96.

#### 2.2.3. Parent–Child Attachment

This study adopted the Parent–Child Attachment Scale (X. Chen et al., 2000), which was adapted from the child attachment behavior classification cards. The scale is a unidimensional measure consisting of 18 items, such as “When your child is upset, he or she actively seeks comfort from you.” Responses were rated on a 5-point Likert scale (1 = never, 5 = always), with some items reverse-coded. Reverse-scored items were first recoded, after which all item scores were summed and then averaged to create a global mean score. Higher scores indicate a more secure level of attachment in preschool children. The scale has demonstrated good reliability and validity in the Chinese cultural context, and the Cronbach’s α coefficient in the present study was 0.76.

#### 2.2.4. Self-Control

This study adopted the short version of the Self-Control Scale (Tangney et al., 2004). The scale is a unidimensional measure consisting of 13 items, such as “It is somewhat difficult for children to concentrate.” Responses were rated on a 5-point Likert scale (1 = never, 5 = always), with some items reverse-coded. Reverse-scored items were first recoded, after which all item scores were summed and then averaged to create a global mean score. Higher scores indicate stronger self-control in young children. The Cronbach’s α coefficient of this scale in the present study was 0.70.

### 2.3. Data Processing and Analysis

Statistical analyses were conducted using SPSS 27.0. First, given the self-report nature of the data, Harman’s single-factor test was performed to assess potential common method bias (Zhou & Long, 2004). Second, descriptive statistics and correlation analyses were conducted to examine the distributions of the study variables and their bivariate associations. Third, after controlling for children’s age and gender, parents’ age and gender, and socioeconomic status (SES), serial regression analyses were conducted to examine the direct associations among parental phubbing, parent–child attachment, children’s self-control, and preschoolers’ excessive electronic media use. Finally, the hypothesized serial mediation model was tested using the PROCESS macro (version 4.1, Model 6) developed by Hayes (2013). This regression-based approach was chosen because it allows for the simultaneous estimation of multiple indirect effects in a theoretically specified sequence while controlling for covariates. To ensure the robustness of the mediation effects, bias-corrected bootstrapping with 5000 resamples was employed to generate 95% confidence intervals. Mediation effects were considered significant when the confidence intervals did not include zero.

## 3. Results

### 3.1. Common Method Bias

This study controlled for common method bias procedurally by employing anonymous measurement and reverse scoring for some questions. According to the common method bias test method (Zhou & Long, 2004), the collected data were subjected to Harman’s single-factor test for common method bias. The unrotated exploratory factor analysis extracted 11 factors with eigenvalues greater than 1. The variance explained by the first factor was 25.97%, which was less than the critical standard of 40%. Therefore, there was no serious common method bias in this study.

### 3.2. Descriptive Statistics and Correlations

Descriptive statistics were first computed for all study variables. Partial correlation analyses were then conducted to examine the associations among parental phubbing, preschoolers’ excessive electronic media use, parent–child attachment, and self-control, while controlling for parents’ gender, parents’ age, child’s gender, child’s age, and socioeconomic status (SES). The results indicated significant associations among these core variables, as presented in Table 1.

### 3.3. Mediating Effect Test

**Figure 2:**
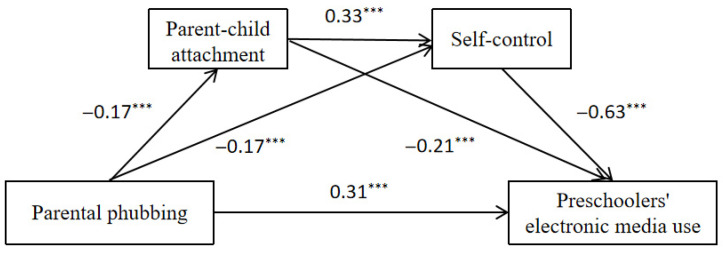
The chain mediating role of parent–child attachment and self-control in parental phubbing and preschoolers’ excessive electronic media use. *** *p* < 0.001.

Using PROCESS 4.1 (Model 6) (Hayes, 2013), we used parental phubbing as the independent variable, preschoolers’ excessive electronic media use as the dependent variable, parent–child attachment and self-control as mediating variables, and parents’ gender, parents’ age, child’s gender, child’s age, socioeconomic status (SES) as control variables. We tested the mediating effect using the bias-corrected nonparametric percentile Bootstrap method (Fang et al., 2012). A 95% confidence interval was calculated based on 5000 repeated samples. The results (as shown in Table 2 and Figure 2) show that parental phubbing significantly and positively predicted preschoolers’ excessive electronic media use (*β* = 0.31, *p* < 0.001), significantly and negatively predicted parent–child attachment (*β* = −0.17, *p* < 0.001) and self-control (*β* = −0.17, *p* < 0.001); parent–child attachment significantly and positively predicted self-control (*β* = 0.33, *p* < 0.001)and significantly negatively predicted preschoolers’ excessive electronic media use (*β* = −0.21, *p* < 0.001); self-control significantly negatively predicted preschoolers’ excessive electronic media use (*β* = −0.63, *p* < 0.001).

The mediation effect analysis (shown in Table 3) shows that the Bootstrap 95% confidence interval of the total indirect effect value of parent–child attachment and self-control does not include 0. This indicates that parent–child attachment and self-control are mediating variables between parental phubbing and preschoolers’ excessive electronic media use. The total mediation effect value is 0.18, accounting for 36.73% of the total effect. Specifically, the mediating effect includes the independent mediating effect of parent–child attachment (effect value 0.04, accounting for 8.16% of the total effect), the independent mediating effect of self-control (effect value 0.11, accounting for 22.45% of the total effect), and the chain mediating role of parent–child attachment and self-control (effect value 0.03, accounting for 6.12% of the total effect). Moreover, the 95% confidence intervals of the three mediating effects do not include 0, indicating that all three mediating effects are significant.

## 4. Discussion

The development of digital technology has made excessive use of electronic media an important public health issue (Sigman, 2012). Preschoolers’ excessive electronic media use has also attracted widespread public attention. Against this backdrop, this study explored the factors influencing preschoolers’ excessive electronic media use and the underlying mechanisms. After controlling for key demographic covariates, the results show that parental phubbing was positively associated with preschoolers’ excessive electronic media use. Furthermore, parent–child attachment and self-control significantly mediated this relation, which contains three mediating pathways—the mediating effects of parent–child attachment and self-control, as well as their chain mediating effect. Overall, these findings enrich the understanding of how parental digital behaviors influence young children’s media use and provide a theoretical basis for developing family-based interventions to promote healthier media habits in preschoolers.

### 4.1. The Relationship Between Parental Phubbing and Preschoolers’ Excessive Electronic Media Use

This study confirms that parental phubbing was associated with preschoolers’ excessive electronic media use (β = 0.31, *p* < 0.001), supporting hypothesis H1. This finding can be explained from the perspective of dynamic two-way interaction. On the one hand, family media norms have a hidden influence on young children. Parental phubbing indirectly forms “family media norms,” and children internalize device operation through observational learning as objects of daily imitation (Bandura, 1977). Young children are significantly more sensitive to nonverbal cues than adolescents (Ding et al., 2018), which may make them more susceptible to the influence of physical gestures such as parental phubbing rather than verbal commands. On the other hand, the dual deprivation of reduced interaction and lack of supervision weakens parent–child emotional bonds (Lauricella & Cingel, 2020) and lowers the threshold for intervention against external temptations (Asplund et al., 2015), jointly driving young children to seek compensation through electronic media. This differs from early models that simply emphasized parental “behavioral modeling” (Bandura, 1977) and highlights the interaction between low autonomy and high environmental dependence in the preschool stage. The results of this study suggest that the physical presence quality of parents during the early childhood stage may have a more substantial impact on behavior shaping than overt educational methods. Overall, the results suggest that the quality of parents’ physical presence in early childhood plays a critical role in shaping young children’s media-related behaviors.

### 4.2. The Mediating Role of Parent–Child Attachment

The results of this study indicate that parent–child attachment can indirectly influence preschoolers’ excessive electronic media use through parental phubbing, verifying hypothesis H2. Specifically, parental phubbing not only deprives preschoolers of emotional responsiveness (David & Roberts, 2017) but also undermines the internalization of attachment security. This, in turn, is associated with lower-quality parent–child interactions (β = −0.17, *p* < 0.001), which may increase young children’s reliance on electronic media. Notably, this finding differs from results reported in studies of adolescents, which have suggested that shared media use may promote intergenerational intimacy (Coyne et al., 2014). This discrepancy may be attributed to developmental differences. In early childhood, family media use typically involves passive consumption, such as watching videos, whereas adolescents are more likely to engage in socially interactive activities, such as cooperative gaming. These differences suggest that the positive effects of “media as family time” may have an age-threshold effect and are less applicable to preschoolers. Recent studies further indicate that when parental electronic media use is accompanied by high-quality language interaction, such as shared reading of electronic picture books, the negative effects of phubbing on parent–child attachment may be partially attenuated (Liu et al., 2024). This finding implies the presence of potential moderating factors within the causal chain linking parental phubbing and attachment impairment.

### 4.3. The Mediating Role of Self-Control

The results of this study indicate that self-control mediates the relationship between parental phubbing and preschoolers’ excessive electronic media use. According to the self-control resource model (He & Shi, 2015), self-control relies on limited psychological resources, and sustained demands on these resources may impair subsequent self-regulatory capacity. Parental phubbing may place additional self-control demands on preschoolers as they attempt to cope with emotional neglect and negative affect, thereby depleting regulatory resources. As a result, the threshold for exerting self-control in later situations—such as resisting excessive electronic media use—may be lowered, increasing the likelihood of maladaptive media engagement. In addition, impaired parent–child interaction may reduce preschoolers’ motivation to internalize parental norms (Y. Sun et al., 2022), further weakening their ability to delay gratification and reinforcing a negative cycle of low self-control and media dependence (Nie et al., 2016). This pattern is consistent with the resource chain depletion mechanism described in prior research (Y. Chen et al., 2023) and highlights the central role of self-control in linking parental phubbing to preschoolers’ excessive electronic media use.

### 4.4. The Chain Mediating Role of Parent–Child Attachment and Self-Control

This study confirmed the chain mediating role of parent–child attachment and self-control in the association between parental phubbing and preschoolers’ excessive electronic media use, supporting Hypothesis 4. This sequential pathway indicates that parental phubbing indirectly increases preschoolers’ excessive media use by undermining parent–child attachment and subsequently weakening self-control. When parents are physically present but emotionally unavailable due to excessive engagement with digital devices, preschoolers may struggle to form secure attachments and internalize behavioral norms (Y. Sun et al., 2022). At the same time, their self-control capacity may be compromised as a result of the continuous depletion of psychological resources. These findings challenge the traditional assumption within attachment theory that physical proximity alone is sufficient to promote emotional bonding (Linder et al., 2021), thereby revealing the increased complexity of parent–child interactions in the digital age. The false sense of emotional connection created by parents’ physical presence may further exacerbate the vulnerability of preschoolers’ self-control systems. Although previous research has demonstrated the positive influence of attachment quality on self-control (M. Wu et al., 2023), the present findings suggest that parental phubbing may simultaneously disrupt emotional connections and deplete self-regulatory resources, making it difficult for traditional regulatory measures, such as behavioral restrictions, to effectively restore impaired self-regulation abilities.

Unlike the single-path effects associated with overprotective or permissive parenting styles, the “quasi-presence” characteristic of parental phubbing is more likely to induce a functional disconnection between emotional regulation and cognitive control. Under sustained emotional strain, preschoolers may fail to internalize behavioral norms through interaction (Nie et al., 2016) and instead develop compensatory dependence on electronic media. This dual deprivation mechanism may help explain why the chain mediating effect observed in this study was significantly stronger than the individual mediating effects. Moreover, the present findings help explain the “efficacy paradox” observed in family digital parenting (Radesky et al., 2014a), suggesting that the effectiveness of interventions depends more on the restoration of emotional connection than on the intensity of monitoring alone. This interpretation echoes prior conclusions regarding the limited effectiveness of purely technological monitoring strategies (R. Sun et al., 2022).

### 4.5. The Role of Demographic Covariates

Several demographic covariates demonstrated significant associations with the core variables in the regression models. First, parents’ gender (1 = father, 2 = mother) was significantly associated with both parent–child attachment and children’s media use. Mothers reported higher attachment quality and lower levels of children’s excessive media use than fathers. This aligns with evidence that mothers typically undertake more daily childcare and emotional management, often adopting more active mediation and monitoring of children’s digital consumption (Craig, 2006; Nikken & Jansz, 2014). Second, parental age was significantly associated with children’s self-control. This finding may reflect age-related differences in parenting experience and emotional maturity, as older parents tend to exhibit more consistent, regulated, and reflective caregiving practices, whereas younger parents may experience higher parenting stress factors that are closely linked to children’s developing self-regulatory capacities (Belsky, 1984). Finally, family socioeconomic status (SES) was significantly associated with children’s self-control. Higher SES is consistently linked to enriched cognitive stimulation and more supportive parenting, which foster better development of self-regulation (Bradley & Corwyn, 2002). Furthermore, families with higher SES tend to possess greater digital maturity and engage in more active parental mediation, such as rule-setting and guided engagement, thereby refining children’s media habits (Koch et al., 2024; Shi et al., 2024). Importantly, although these covariates showed independent effects, their inclusion did not alter the significance or pattern of the hypothesized mediation pathways.

### 4.6. Implications

This study explored the mechanisms of preschoolers’ excessive electronic media use and provided preliminary insights for interventions to reduce excessive use of electronic media among preschoolers. Based on the four pathways which parental phubbing influences preschoolers’ excessive electronic media use, a layered intervention strategy can be attempted. (1) Direct intervention in parental phubbing. Use functional apps to enable “focus mode” on mobile phones during parent–child interaction times such as meals and games, automatically blocking non-urgent notifications (Radesky et al., 2014a). Future intervention efforts may consider exploratory dual-assessment approaches to “physical presence–emotional presence,” such as using wearable technologies to capture patterns of parental gaze diversion and provide gentle feedback. Encourage “shared media use” methods such as reading electronic picture books together and transform passive viewing into interactive learning through guided participation (Liu et al., 2024) to offset the negative effects of phubbing. (2) Intervention in the quality of parent–child relationships. In response to the reduction in eye contact and interaction caused by phubbing (Kildare & Middlemiss, 2017). One possible approach is to introduce simple, time-based response guidelines for parents, such as a “3-3-3 response rule”. When children send interaction signals, parents should look up within 3 s, maintain eye contact for 3 s, and describe the child’s behavior in 3 sentences. Try “attachment repair time”: Set aside 20 min daily for “highly responsive time” free from media distractions. Parents should synchronously mimic the child’s nonverbal actions (e.g., facial expressions, gestures) to reinforce the experience of a secure base. (3) Train preschoolers in self-control. Introduce “stepwise delayed gratification” tasks: When children become anxious because their parents are phubbing, provide progressive waiting rewards (e.g., “After Mom finishes this message, we can tell one more story”). This type of training can enhance emotional regulation resilience through resource restoration mechanisms (Inzlicht et al., 2014). (4) Intervention in the chain effect of parent–child attachment and self-control. Design a “media transition ritual” in which parents establish a five-minute connection buffer (e.g., hugging, repeating the child’s current activity) before ending their cell phone use. Such rituals can reduce self-control resource depletion through attachment pre-repair (Radesky et al., 2014a; Y. Sun et al., 2022). In summary, effective interventions need to go beyond simply limiting screen time. Through a three-dimensional integration of technological intervention, emotional connection, and cognitive restructuring, the challenges of parent–child interaction in the digital age may be reframed as opportunities to support the development of self-control, thereby reducing excessive use of electronic media among preschoolers.

### 4.7. Limitations

Several limitations must be acknowledged. First, all variables in this study were assessed using parent self-report measures, which introduces the possibility of systematic reporting inaccuracies. Parents may underestimate or overestimate their own smartphone use, their children’s electronic media use, as well as relational or self-regulatory characteristics such as parent–child attachment and children’s self-control. Although objective assessments of children’s electronic media use (e.g., device-based logs or usage tracking) are often difficult or costly to implement in large-scale field studies, future research should seek to incorporate multi-informant reports (e.g., child or teacher reports) and multi-method approaches to improve measurement accuracy and validity. Second, the sample size of this study was determined by the study design and participant availability rather than by an a priori power analysis. Although the relatively large sample enhances statistical stability, it may also increase the likelihood of detecting statistically significant effects with small effect sizes. Future research is encouraged to conduct a priori power analyses to inform sample size planning and to further validate the robustness of the observed associations. Third, this study did not directly assess the specific number or types of electronic devices in the home, nor did it measure specific parental mediation rules (e.g., time limits or content restrictions). While family SES was controlled for as a proxy for digital resource accessibility, future research should incorporate more granular measures of the home media environment and household electronic availability to further refine the findings. Finally, the present study adopted a cross-sectional design. Although the statistical indices and theoretical framework support the hypothesized model, it is inherently impossible to establish definitive causal relationships. There may be bidirectional influences or reciprocal feedback loops among the variables. Future research should consider employing longitudinal designs or experimental methods to further establish causal directions and verify the stability of this developmental trajectory.

## 5. Conclusions

This study explored the psychological mechanisms underlying preschoolers’ electronic media use in the digital context. The results demonstrate that parental phubbing is positively associated with excessive electronic media use among preschoolers. Furthermore, the relationship is partially mediated by both parent–child attachment and children’s self-control. Most importantly, the study identifies a significant chain mediating pathway, suggesting that parental phubbing undermines the quality of parent–child attachment, which in turn weakens children’s self-control, ultimately leading to increased risks of excessive media use.

## Figures and Tables

**Table 1:** Descriptive statistics and correlation analysis between variables (N = 758).

	*M* ± *SD*	1	2	3	4
1 Parental phubbing	2.53 ± 0.58	1			
2 Preschoolers’ excessive electronic media use	2.06 ± 0.78	0.37 ***	1		
3 Parent–child attachment	3.79 ± 0.50	−0.19 ***	−0.33 ***	1	
4 Self-control	3.33 ± 0.46	−0.29 ***	−0.50 ***	0.40 ***	1

Note. *** *p* < 0.001. The same applies below. Partial correlations controlling for parents’ gender, parents’ age, child’s gender, child’s age, and SES.

**Table 2:** Regression analysis of variable relationships in the model.

Regression Equation		Overall Fit Index			Significance of the Regression Coefficient	
Outcome variable	Predictor variable	*R*	*R* ^2^	*F*	*β*	*t*
Parent–child attachment	Parental phubbing	0.23	0.05	7.13	−0.17	−5.39 ***
	Parents’ gender				0.11	2.50 *
	Parents’ age				0.01	1.59
	Child’s gender				0.01	0.37
	Child’s age				0.02	0.95
	SES				0.01	1.03
self-control	Parental phubbing	0.49	0.24	33.40	−0.17	−6.67 ***
	Parent–child attachment				0.33	10.76 ***
	Parents’ gender				0.01	0.31
	Parents’ age				0.01	2.20 *
	Child’s gender				0.01	0.47
	Child’s age				0.02	1.15
	SES				0.03	3.69 ***
Preschoolers’ excessive electronic media use	Parental phubbing	0.58	0.34	47.91	0.31	7.41 ***
	Parent–child attachment				−0.21	−4.18 ***
	Self-control				−0.63	−11.06 ***
	Parents’ gender				−0.16	−2.70 **
	Parents’ age				0.01	1.12
	Child’s gender				−0.06	−1.26
	Child’s age				0.01	0.42
	SES				−0.01	−0.84

Note: All variables were standardized before being entered into the regression equation. Analysis controlled for parents’ gender, parents’ age, child’s gender, child’s age, and SES. * *p* < 0.05, ** *p* < 0.01, *** *p* < 0.001.

**Table 3:** Analysis of the mediating effect of parent–child attachment and self-control.

Path	Effect Value	Boot Standard Error	Boot CI Lower Limit	Boot CI Upper Limit	Relative Intermediary Effect
Total effect	0.49	0.04	0.40	0.58	
Direct effect	0.31	0.04	0.23	0.39	
Total indirect effect	0.18	0.03	0.13	0.24	36.73%
Parental phubbing ⟶ Parent–child attachment ⟶ Excessive electronic media use	0.04	0.01	0.02	0.06	8.16%
Parental phubbing ⟶ Self-control ⟶ Excessive electronic media use	0.11	0.02	0.07	0.16	22.45%
Parental phubbing ⟶ Parent–child attachment ⟶ Self-control ⟶ Excessive electronic media use	0.03	0.01	0.02	0.05	6.12%

Note: Standard error, lower limit, and upper limit refer to the standard error, lower limit, and upper limit of the 95% confidence interval of the indirect effect estimated by the percentile Bootstrap method with deviation correction, respectively.

## Data Availability

All data included in this study are available upon request by contacting the corresponding author.
